# Nanosecond pulsed electric fields enhanced chondrogenic potential of mesenchymal stem cells via JNK/CREB-STAT3 signaling pathway

**DOI:** 10.1186/s13287-019-1133-0

**Published:** 2019-01-24

**Authors:** Tong Ning, Jinsong Guo, Kun Zhang, Kejia Li, Jue Zhang, Zheng Yang, Zigang Ge

**Affiliations:** 1 Peking-Tsinghua Center for Life Sciences, Beijing, 100871 China; 2 Department of Biomedical Engineering, College of Engineering, Peking University, Beijing, 100871 China; 3 Institute of Biomechanics and Biomedical Engineering, College of Engineering, Peking University, Beijing, 100871 China; 4 Center for BioMed-X Research, Academy for Advanced Interdisciplinary Studies, Peking University, Beijing, 100871 China; 5 Academy for Advanced Interdisciplinary Studies, Peking University, Beijing, 100871 China; 6 Tissue Engineering Program, Life Sciences Institute, National University of Singapore, 27 Medical Drive, Singapore, 117510 Singapore

**Keywords:** Pulsed electric fields, Stem cells, Electrical stimulation, Chondrogenic differentiation, Cartilage regeneration

## Abstract

**Background:**

Nanosecond pulsed electric fields (nsPEFs) can produce more significant biological effects than traditional electric fields and have thus attracted rising attention in developing medical applications based on short pulse duration and high field strength, such as effective cancer therapy. However, little is known about their effects on the differentiation of stem cells. Furthermore, mechanisms of electric fields on chondrogenic differentiation of mesenchymal stem cells (MSCs) remain elusive, and effects of electric fields on cartilage regeneration need to be verified in vivo. Here, we aimed to study the effects of nsPEFs on chondrogenic differentiation of MSCs in vitro and in vivo and further to explore the mechanisms behind the phenomenon.

**Methods:**

The effects of nsPEF-preconditioning on chondrogenic differentiation of mesenchymal stem cells (MSCs) in vitro were evaluated using cell viability, gene expression, glycosaminoglycan (sGAG) content, and histological staining, as well as in vivo cartilage regeneration in osteochondral defects of rats. Signaling pathways were investigated with protein expression and gene expression, respectively.

**Results:**

nsPEF-preconditioning with proper parameters (10 ns at 20 kV/cm, 100 ns at 10 kV/cm) significantly potentiated chondrogenic differentiation capacity of MSCs with upregulated cartilaginous gene expression and increased matrix deposition through activation of C-Jun NH2-terminal kinase (JNK) and cAMP-response element binding protein (CREB), followed by activation of downstream signal transducer and activator of transcription (STAT3). Implantation of nsPEF-preconditioned MSCs significantly enhanced cartilage regeneration in vivo, compared with implantation of non-nsPEF-preconditioned MSCs.

**Conclusion:**

This study demonstrates a unique approach of nsPEF treatment to potentiate the chondrogenic ability of MSCs through activation of JNK/CREB-STAT3 that could have translational potential for MSC-based cartilage regeneration.

**Electronic supplementary material:**

The online version of this article (10.1186/s13287-019-1133-0) contains supplementary material, which is available to authorized users.

## Introduction

Electric field stimulations, both intrinsic and extrinsic, are involved in directing stem cell fate, by directly acting either on the cells and/or through cell niches [1, 2]. However, limited with relative long duration (above microseconds) and low field strength (around V/cm), these electrical stimulations usually cast weak and non-specific biological effects on cells, mainly on the cell membrane [3]. Diversity of parameters and off-the-target effects further limit their biomedical applications [4].

Nanosecond pulsed electric fields (nsPEFs) with short duration (nanoseconds) and high field strength (above thousand V/cm) are able to instigate focused biological effects on cells beyond the plasma membrane and can produce more focused biological effects than traditional electric fields [5]. Application of nsPEFs is associated with formation of nanopores in cell membranes, transient release of calcium ions [6], and selective effects on intracellular structures and their functions [7]. The degree of the outer membrane permeabilization to that of subcellular membranes is affected by the pulse parameters, such as pulse duration, the rise time, pulse amplitude and number, and even pulse repetition rate, which depends on cell type. High intensity nsPEFs, with the capacity to induce necrosis [8] or apoptosis in mammalian cells [9] in part through mitochondrial and caspase-dependent mechanisms [10], and to reduce tumor size in vivo, have been explored as an emerging cancer therapy [11]. At low intensity, nsPEFs caused calcium mobilization from intracellular structures [12] and could impart diverse biological effects, such as the enhancement of chondrocyte proliferation and dedifferentiation [13], proliferation of endothelial cells [14], damage-free excitation of peripheral nerve [15], and cardiomyocytes [16]. The intracellular mechanisms induced by nsPEFs need further investigation. On Hela cells, nsPEFs incurred strong and transient activation of the mitogen-activated protein kinase (MAPK) pathway, including p38, JNK, and ERK and their upstream kinases [17]. nsPEFs were also reported to affect cell differentiation by triggering oxidative burst in algae cells [18]. In our previous study, we showed that nsPEFs regulated phenotypes of primary chondrocytes through selectively activating Wnt/β-catenin pathways [13].

**Fig. 1:**
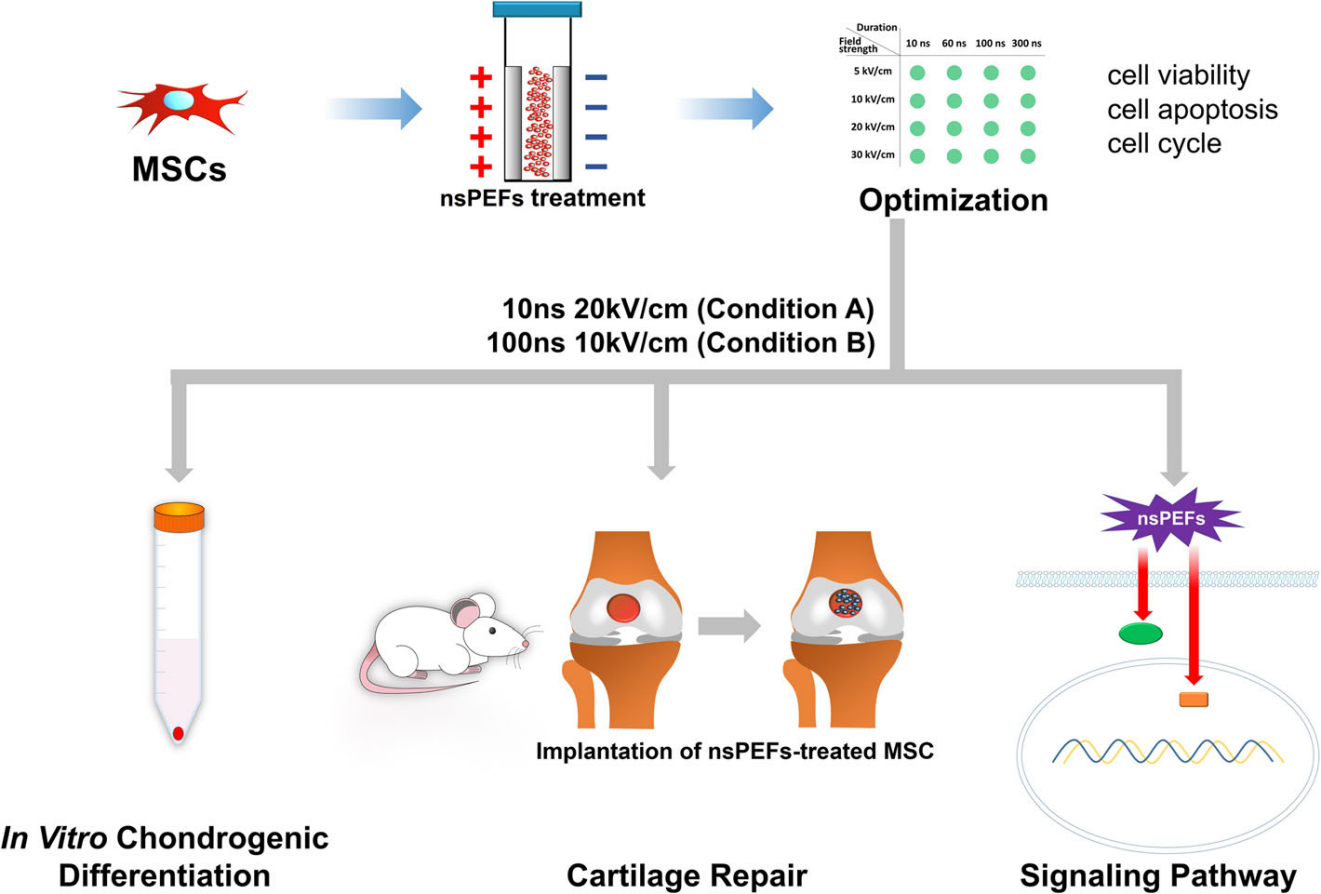
Schematic illustration of the experimental setup

The effects of electrical stimulations on chondrogenic differentiation of mesenchymal stem cells (MSCs) are still unclear. A few studies have examined the effect of traditional pulsed electrical fields [19–21], in which induction of Sox5, Sox6, and p-ERK1/2 [19] and a dependence on Ca^2+^/ATP signaling [20] have been implicated in promoting chondrogenic differentiation. However, the mechanisms, and their effectiveness need to be unregulated in vitro and verified in vivo. In this study, instead of applying electric pulse at intermittent periods during the differentiation period, we took the approach of subjecting MSCs to nsPEFs prior to chondrogenic induction, with the objective of investigating the preconditioning effect of nsPEFs to potentiate subsequent differentiation. We first characterized the effects of nsPEFs, by varying the pulse intensity and duration parameters, on MSC viability and apoptosis, as well as chondrogenic gene expression after the preconditioned cells were subjected to differentiation procedure. The ability of nsPEF-preconditioned MSCs to regenerate cartilage in vivo was validated using a rat cartilage defect model. The involvement of intracellular signaling pathways activated by nsPEFs was also explored (Fig. 1).

## Materials and methods

### Cell culture

Porcine bone marrow-derived stromal cells (MSCs), an attractive cell source for cell therapy in cartilage [22], were harvested from femur and tibia of 6- to 10-month-old pigs. The collected cells were washed with phosphate-buffered saline (PBS) and then cultured in a monolayer culture in medium containing Dulbecco’s modified Eagle’s medium (DMEM, Gibco), 10% fetal bovine serum (FBS, Gibco), and 0.1% penicillin/streptomycin (PS, Amresco) at 37 °C with 5% CO_2_. Non-attached cells were removed after a week, and adherent cells were allowed to expand further in medium containing DMEM and 10% FBS at 37 °C with 5% CO_2_. The medium was changed twice weekly. MSCs were trypsinized with 0.25% trypsin (Invitrogen) at around 85% confluency and further expanded. MSCs at passages 4 and 5 were used for all subsequent experiments. BI-78D3 (10 μmol/L, SIGMA), BAPTA-AM (10 μmol/L, Invitrogen), and Stattic (10 μmol/L, targetmol) were used to inhibit the phosphorylation of JNK, CREB, and STAT3, respectively.

### Application of nsPEFs

One million MSCs suspended in 1 mL of PBS within a 0.4-cm gap cuvette (BTX electroporation cuvette #45–0125) for 5–20 kV/cm, and a 0.2-cm gap cuvette (#45–0126) for 30 kV/cm, were subjected to electric field with durations of 10 ns, 60 ns, 100 ns, or 300 ns, as previously described [13]. Five pulses were applied at 1-s intervals in between each pulse. MSCs without nsPEF stimulation served as the control group.

### Chondrogenic differentiation of mesenchymal stem cells in pellet culture

After electrical stimulation, 2.5 × 10^5^ MSCs were suspended in chondrogenic medium consisting of high-glucose DMEM with 10 ng/mL transforming growth factor-β3 (TGF-β3, Sigma), 100 nM dexamethasone (Sigma), 1% insulin–transferrin selenium premix (ITS, Gibco), 1 mM sodium pyruvate (Sigma), 50 mg/mL L-proline (Sigma), and 50 mg/mL ascorbate-2-phosphate (Sigma). MSCs were centrifuged at 500*g* for 5 min to form a pellet in 15-mL conical polypropylene tubes (Corning). The medium was changed every 3 days, and the pellets were harvested at day 14.

### Viability of the cell

Viability of the cells was evaluated with Cell Counting Kit-8 (CCK-8 assay, Sigma) at days 1 and 3 after nsPEF treatment. Briefly, 10 microliters of CCK-8 solution were added to each well and incubated for 1–4 h. The absorbance was measured at a wavelength of 450 nm using the Microplate Reader (680, Bio-Rad). The reference wavelength was set at 600 nm. The value was expressed as the ratio of the experimental absorbance over the control (non-nsPEF treatment) absorbance. Four samples from each group were measured.

### Apoptosis of the cells

Apoptosis of the cells was analyzed after 1 h of nsPEF treatment with Annexin V-FITC/propidium iodide (PI) Apoptosis Detection Kit according to the manufacturer’s protocol. Cells were collected using trypsin without EDTA and washed with calcium-free PBS, then resuspended in binding buffer. Annexin V-FITC was added to the suspension and incubated at room temperature for 15 min. PI was added to the suspension 5 min before the analysis. The distribution of Annexin V-FITC and PI-positive cells was analyzed with the BD FACSCalibur Flow Cytometer, and the fold changes of live cells were presented relative to the non-nsPEF-preconditioned control samples.

### Gene expression

Total RNA was extracted from pellets or cells in each culture condition with Trizol Reagent (New Industry) following the manufacturer’s protocol. Total RNA was quantified with the Nanodrop Spectrophotometer (ND-1000, Thermo), and the reverse transcription reaction was performed on 1000 ng of RNA as previously described [13]. Quantitative real-time polymerase chain reactions (PCR) were performed on a Pikoreal 96 PCR System (Thermo) following the manufacturer’s procedures. The expression of type I collagen (*COL I*), type II collagen (*COL II*), type X collagen (*COL X*), aggrecan (*ACAN*), and *SOX9* were analyzed with qRT-PCR with the gene-specific primers listed in Additional file 1: Table. S1. The target genes of each sample were normalized to the values of glyceraldehyde-3-phosphate dehydrogenase (GAPDH) as internal control. Relative expression of each gene was expressed as fold changes by the 2^−ΔΔCt^ method. Five samples of each group were measured. Statistical significance was marked with different letters (*P* < 0.05).

### Histology

At day 14 of chondrogenic differentiation, the pellets were fixed in 4% paraformaldehyde, followed by dehydration in the ascending series of ethanol, washed in xylene, and embedded in paraffin, then cut into 5-μm sections. Alcian blue was used to stain sulfated glycosaminoglycan (sGAG); hematoxylin and eosin (H&E) were used to stain the nuclei, cytoplasm, and extracellular matrix. Safranin O/fast green was used to stain mature cartilage. The samples were then observed under a microscope.

### Sulfated glycosaminoglycan (sGAG) quantification

At day 14 of chondrogenic differentiation, pellets were digested in proteinase K at 56 °C for 12 h, and the digested solution was mixed with dimethylmethylene blue (DMMB, Sigma) working solution as previously described [13]. The mixed solution was shaken for 30 min, and then centrifuged at 10,000*g* for 10 min. The decomplexation solution was added to dissolve the centrifugal sediment and absorbance was measured at 630 nm. Five samples of each group were measured.

### Western blotting

Cells after nsPEF stimulation were collected at 0.5 h and lysed by RIPA lysis buffer (R0020, Solarbio). The western blotting was performed according to the manufacturer’s protocol [13]. Rabbit polyclonal antibodies against Phospho-P38 MAPK (4511, Cell Signaling), P38 MAPK (8690, Cell Signaling), ERK1/2 MAPK (4695, Cell Signaling), Phospho-ERK1/2 MAPK (4370P, Cell Signaling), JNK MAPK (9252, Cell Signaling), Phospho-JNK MAPK (4668, Cell Signaling), CREB (4820, Cell Signaling), Phospho-CREB (9198, Cell Signaling), STAT3 (4904, Cell Signaling), Phospho-STAT3 (9145, Cell Signaling), β-catenin (sc-7199, Santa Cruz Biotechnology), and β-actin (13E5, Cell Signaling) were utilized to detect the targeted proteins, followed by incubation with secondary HRP-linked antibody of anti-rabbit IgG (Cell Signaling). The complex of the antigen and the antibody was detected with TANON 1600 Gel Imaging System, and the expression level of protein is analyzed with Tanon Gis. Statistical significance was marked with different letters (*P* < 0.05).

### Regeneration of articular cartilage defects

All animal experiments were conducted according to the Institutional Animal Care and Use Committee of Peking University. Ten-week-old male SD rats were used. Articular cartilage defects were created in both knees of the rats. Prior to surgery, each animal was anesthetized intraperitoneally with 10% (*w*/*v*) chloral hydrate (23100, Sigma) dissolved in PBS buffer without calcium. Defects (1.5 mm in depth and 1.5 mm in diameter) were created in the center of articular cartilage of the femoral trochlea. MSCs were stimulated with nsPEFs under selected parameters. Three hundred thousand of the nsPEF-treated MSCs, constituted in 10 μL of alginate (1% *v*/*v*), were then injected into the defects. Ten microliters of calcium chloride (10% *w*/*v*) were added to form a gel with the alginate. The control group was implanted with MSCs without nsPEF treatment. After closure, 200 μL of chondrogenic differentiation medium containing TGF-β3 (10 ng/mL) was injected into the joint cavity of both knees. Rats (*n* = 6 per group) were sacrificed at 12 weeks after surgery. The whole knee joints were fixed in 10% formalin for 24 h at 4 °C. The tissues were decalcified with 10% (*w*/*v*) EDTA for 2 weeks and embedded in paraffin. Subsequently, sections of 5-μm thickness through the center of the defect were prepared for histological and immunohistochemistry staining.

### Immunohistochemistry

Monoclonal antibodies specific for collagen type I (ab23446, Abcam), collagen type II (ab34712, Abcam), and collagen type X (ab49945, Abcam) were applied, and the mixture was incubated at 4 °C overnight, followed by addition of biotinylated goat anti-mouse or rabbit anti-goat secondary antibodies. The tissues were scored using the ICRS Macroscopic score assessment and ICRS Visual Histological Assessment Scale.

### Statistical analysis

Data was presented as mean ± SD and was normalized to the control group, defined as 1. One-way ANOVA analysis was carried out with the least significant difference (LSD) test using SPSS 13.0 software (SPSS Inc.). The statistical significance level was set as *P* < 0.05.

## Results

### Optimization of parameters of nsPEFs

**Fig. 2:**
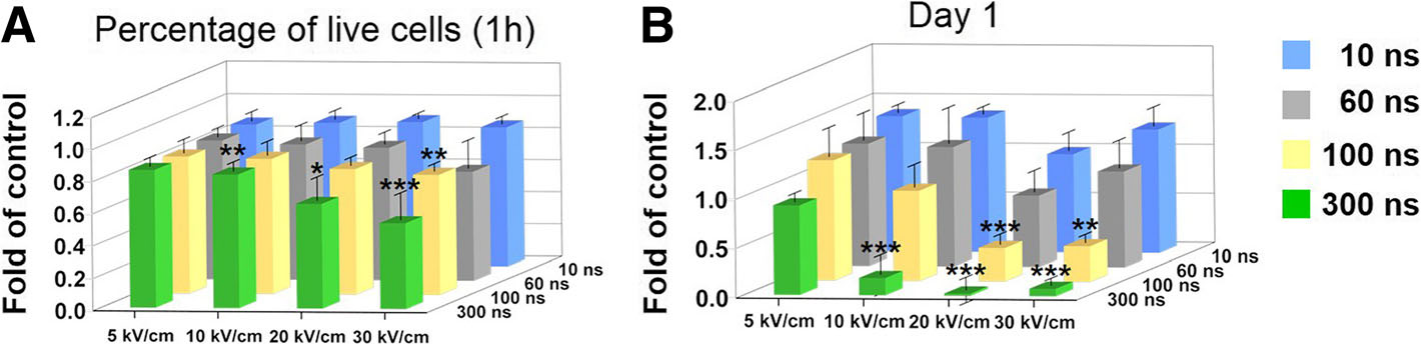
Effects of nsPEFs on MSC apoptosis and viability. **a** Fold changes of live cells by flow cytometry of Annexin V-FITC/PI double stained cell, compared with the control group (non-pulsed samples). *n* = 4. **b** Cell count as quantified by Kit-8 assay at day 1. *n* = 4 for each group. Cell count was expressed as fold of control (non-pulsed samples). *, *P* < 0.05; **, *P* < 0.01; ***, *P* < 0.001

To optimize the parameters, cytotoxic effects of nanosecond pulsed electric fields (nsPEFs) with 16 conditions (4 durations with 4 field strength) on the MSCs were evaluated. No significant apoptosis was observed, indicated by the presence of ~ 80% live cells, at 1 h after nsPEF treatment in a majority of the 16 nsPEF conditions, except 300 ns at 20 kV/cm and 60, 100, and 300 ns at 30 kV/cm, compared with the non-nsPEF-preconditioned cells (Fig. 2a). However, a significant decrease in cell viability was detected at day 1 after nsPEFs with increased pulse duration and field strength, with the percentage of viable cells dropping to less than 40%. The percentage of viable cells decreased drastically at 20 and 30 kV/cm with longer pulse duration while nsPEFs at 5 kV/cm showed little toxic effects even with longer pulsing time (Fig. 2b). Ten conditions that had no toxic effects were utilized to evaluate their effects on chondrogenic differentiation of MSCs described in the next section.

### Effects of nsPEF-preconditioning on chondrogenic differentiation

**Fig. 3:**
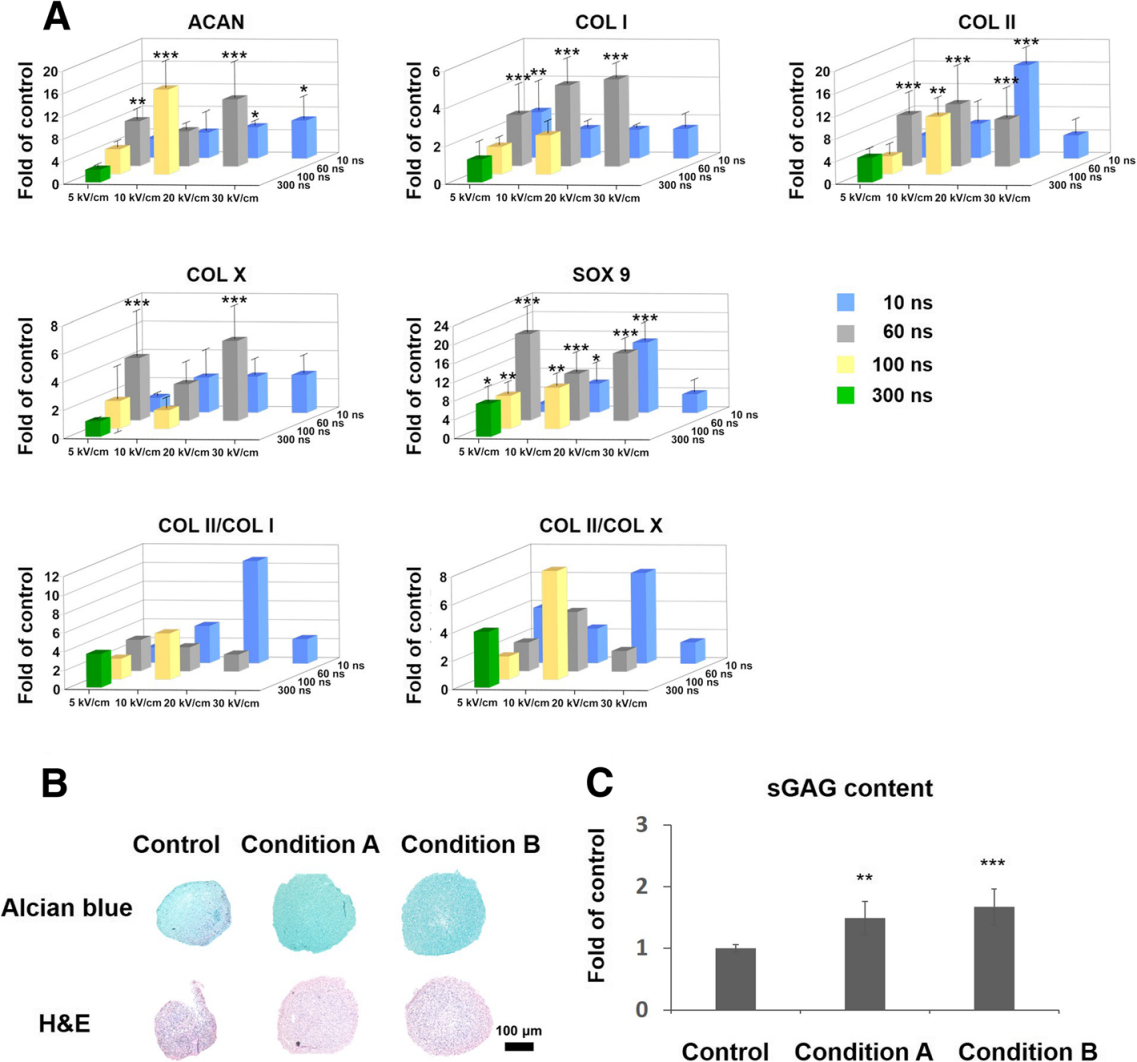
Pre-conditioning with nsPEFs promoted chondrogenic differentiation *of MSCs* in vitro. **a** Expression level for *SOX9*, *COL II*, *COL I*, *COL X*, *ACAN*, and *COL II/I*. *n* = 5 for each group, compared with the control group (non-pulsed samples). The data was normalized by setting the non-nsPEFs control group as 1. **b** Alcian blue staining (upper) and H&E staining (lower) in pellets. Representative of per group (*n* = 4). **c** sGAG quantitation in pellets, *n* = 5 per group

MSCs in suspension were exposed to varying pulsing parameters and immediately subjected to chondrogenic differentiation in 3D pellet format for 14 days. nsPEF preconditioning of 10 ns at 20 kV/cm (condition A) and 100 ns at 10 kV/cm (condition B) significantly increased gene expression levels of *COL II*, *SOX9*, and *ACAN* ranging from about 5 to 16 folds compared with the non-nsPEF-preconditioned cells (Fig. 3a). The expression level of fibro and hypertrophy genes (*COL I* and *COL X*) was measured, and the calculated *COL II*/*COL I* ratio and *COL II*/*COL X* ratio indicate enhancement with 10 ns at 20 kV/cm and 100 ns at 10 kV/cm, compared with the non-nsPEF-preconditioned cells. Although nsPEF preconditioning of 60 ns at 5 kV/cm, 10 kV/cm, or 20 kV/cm also resulted in significant upregulation of *COL II*, *SOX9*, and *ACAN*, these conditions concomitantly upregulated *COL I* and/or the hypertrophy marker, *COL X*. The other combinations of parameters exerted weaker effects on chondrogenic genes. The results suggest that nsPEF preconditioning with specific parameters (conditions A and B) may promote optimum chondrogenic differentiation of MSCs for the formation of hyaline cartilage, without triggering hypertrophy during the process.

We further evaluated the chondrogenic differentiation potential of nsPEF-preconditioned MSCs with histological staining and sulfated glycosaminoglycan (sGAG) quantification. nsPEFs of both 10 ns at 20 kV/cm (condition A) and 100 ns at 10 kV/cm (condition B) upregulated the sGAG formation compared with the control group, indicated by Alcian blue staining (Fig. 3b). The quantitated amount of sGAG increased by 1.49- and 1.67-fold with conditions A and B, respectively, relative to the non-nsPEF-preconditioned control group (Fig. 3c). Taken together, in vitro results indicate that preconditioning with nsPEFs using condition A and condition B could promote subsequent chondrogenic differentiation of MSCs in vitro.

### Regeneration of articular cartilage defects

**Fig. 4:**
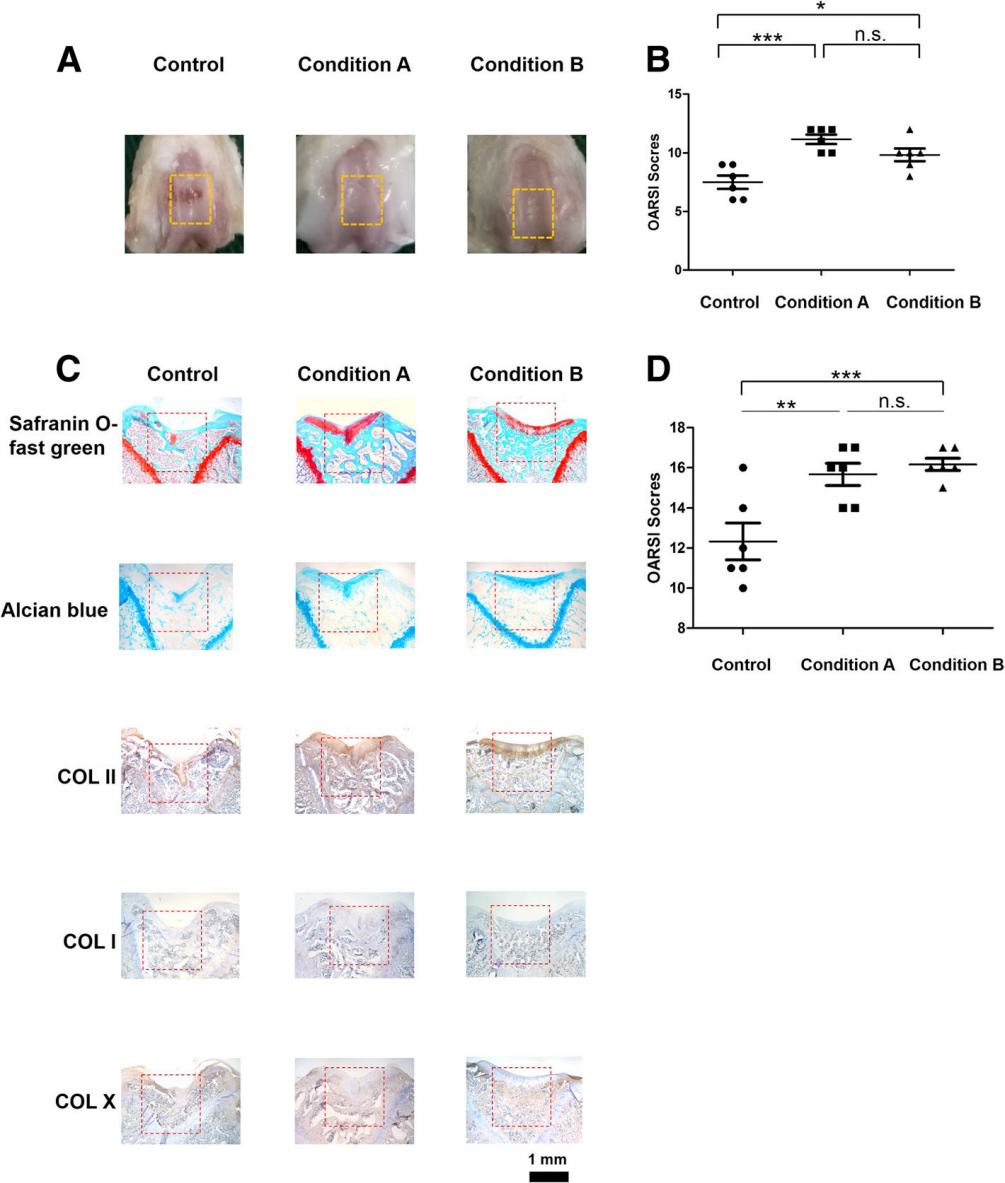
nsPEF-preconditioned MSCs enhanced cartilage regeneration in vivo. **a** Macroscopic view of cartilage repair. **b** ICRS Macroscopic score of joint. *n* = 6 per group. **c** Safranin o-fast green staining, Alcian blue staining, immunohistochemical staining of sections for collagen type II, collagen type I and collagen type X. **d** ICRS visual histological score of tissue sections. *n* = 6 per group

To evaluate the ability of nsPEF-preconditioned MSCs in cartilage regeneration in vivo, MSCs were exposed to conditions A and B and implanted into rat osteochondral defects. At 12 weeks post-surgery, defects implanted with nsPEF-preconditioned MSCs (both conditions A and B) showed a smoother cartilage surface than the control group implanted with non-nsPEF-preconditioned MSCs (Fig. 4a). The overall repair assessment by ICRS macroscopic score (Fig. 4b) and ICRS visual histological score (Fig. 4d) showed implantation of MSCs after nsPEF treatment yielded higher scores compared with non-nsPEF-preconditioned MSCs. There were no significant differences between the two nsPEF treatment groups. Collagen II, Alcian blue, and Safranin O staining were stronger in the nsPEF-preconditioned groups, while no significant collagen I or collagen X staining was detected (Fig. 4c).

### nsPEFs promote phosphorylation of JNK, CREB, and STAT3

**Fig. 5:**
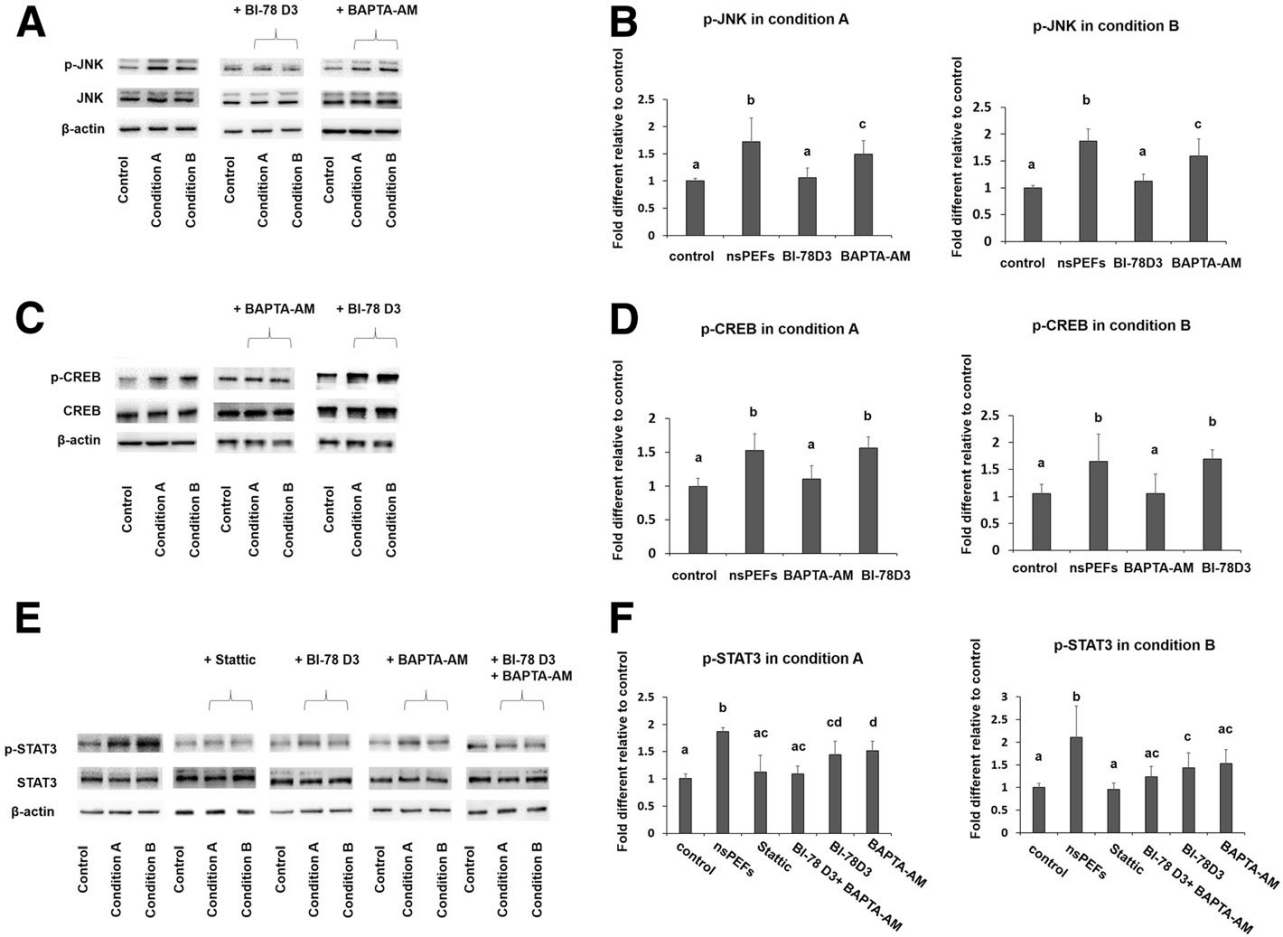
nsPEFs activated JNK, CREB, and STAT3 signaling pathways. **a** Western blot analysis for JNK, p-JNK with inhibitors for phosphorylation of JNK (BI-78D3), and CREB (BAPTA-AM). **b** Phosphorylation ratio for JNK with inhibitors, *n* = 5. **c** Western blot analysis for CREB, p-CREB with inhibitors for phosphorylation of JNK (BI-78D3), and CREB (BAPTA-AM). **d** Phosphorylation ratio for CREB with inhibitors, *n* = 5. **e** Western blot analysis for STAT3, p-STAT3 with inhibitors for phosphorylation of JNK (BI-78D3), CREB (BAPTA-AM), and STAT3 (Stattic). **f** Phosphorylation ratio for STAT3 with inhibitors, *n* = 5. Statistical significance in mean values was marked with different letters. For all variables with the same letter (**a**, **b**, **c**, **d**, or **e**), the difference between the groups is not statistically significant. For variables with a different letter, the difference between the groups is statistically significant (*P* < 0.05)

To study the mechanism of nsPEF-potentiated chondrogenic differentiation, various signaling pathways previously implicated in electric stimulation and MSC differentiation were investigated, including JNK, P38, ERK, Wnt, CREB, and STAT3. nsPEFs (conditions A and B) resulted in upregulated phosphorylation of JNK, CREB, and STAT3, with no effect on the P38, ERK, and Wnt signaling pathways after 1/2 h post-nsPEF treatment (Fig. 5a, c, e and Additional file 2: Figure S2). The expression level of c-Jun, which is downstream of JNK, increased at 2 h after nsPEF treatment (Additional file 3 Figure S1B).

### Inhibition of phosphorylation of JNK, CREB, or STAT3 hindered chondrogenic differentiation induced by nsPEF preconditioning

**Fig. 6:**
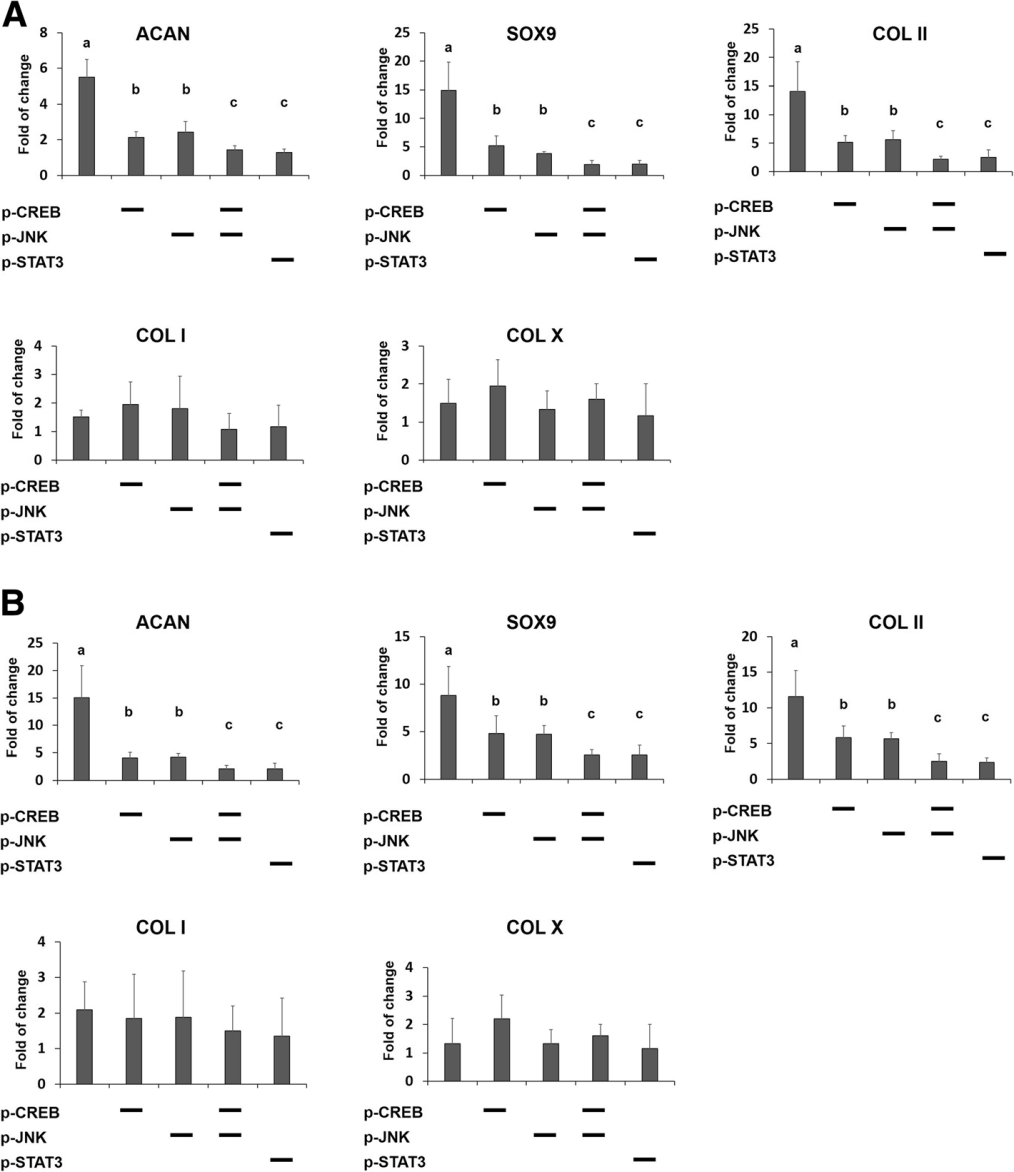
nsPEFs promoted MSC chondrogenic differentiation through JNK/CREB-STAT3 signaling pathway. Expression levels for *SOX9*, *COL II*, *COL I*, *COL X*, and *ACAN* in the absence or presence of inhibitors of either phosphorylation of CREB, JNK, or STAT3, or combination of them with (**a**) condition A, 10 ns at 20 kV/cm, and (**b**) condition B, 100 ns at 10 kV/cm. Diagonal (−) means inhibitors for corresponding proteins. Statistical significance in mean values was marked with different letters

To confirm that JNK, CREB, or STAT3 activation is involved in chondrogenic differentiation induced by nsPEF preconditioning, the effects of phosphorylation inhibition of JNK, CREB, and STAT3 were investigated. Enhanced phosphorylation of JNK, CREB, and STAT3 induced by nsPEFs were inhibited by the specific inhibitors BI-78D3, BAPTA-AM, and Stattic, respectively(Fig. 5), which in turn inhibited the expression of *SOX9*, *COL II*, and *ACAN* induced by nsPEF preconditioning under condition A (Fig. 6a) or condition B (Fig. 6b). Inhibition of either JNK or CREB phosphorylation could reduce the expression level of *SOX9*, *COL II*, and *ACAN* caused by nsPEFs to about 30–50%, while combined inhibition of JNK together with CREB could further reduce the expression level by another 50% relative to the singular inhibitor treatment (Fig. 6a, b). Notably, inhibition of STAT3 phosphorylation alone reduced the expression of *SOX9*, *COL II*, and *ACAN* to similar levels comparable to the combined inhibition of JNK and CREB.

**Fig. 7:**
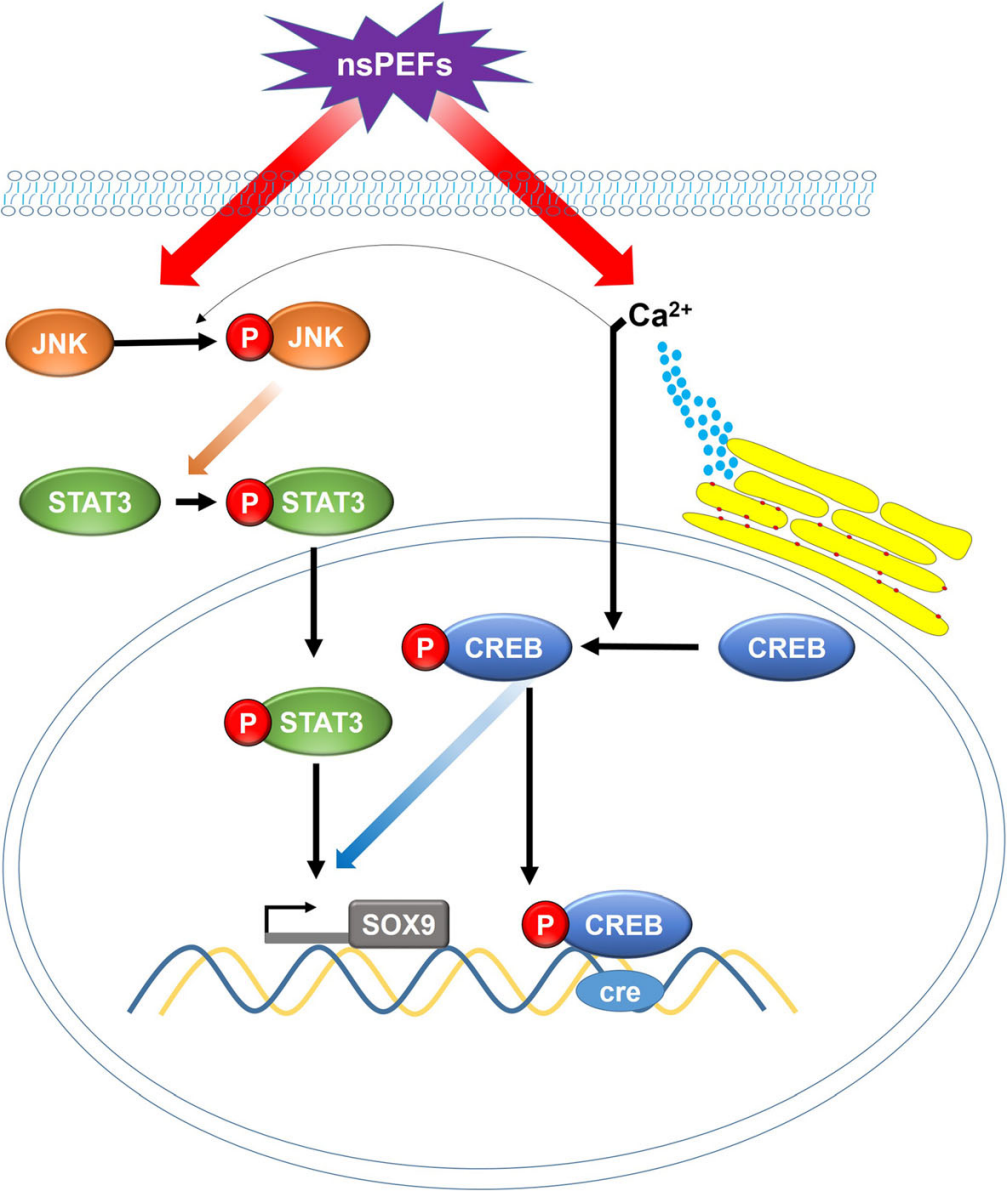
Schematic illustration of the possible signaling pathways induced by nsPEFs in MSCs

The possibility of cross talk between the JNK, CREB, and STAT3 pathways was examined. Inhibition of CREB phosphorylation with BAPTA-AM, a calcium chelator, slightly affected the upregulated phosphorylation of JNK by nsPEFs (Fig. 5a, b). Inhibition of JNK phosphorylation with BI-78D3 did not affect the upregulated phosphorylation of CREB (Fig. 5c, d). On the other hand, inhibition of JNK or CREB phosphorylation alone downregulated the phosphorylation of STAT3 induced by nsPEFs (Fig. 5e, f), suggesting that JNK and CREB were upstream regulators of STAT3 signaling; intracellular calcium may play a role in the activation of JNK signaling pathways. These results suggest that nsPEF preconditioning promotes chondrogenic differentiation of MSCs through activation of JNK and CREB, and is followed by activation of downstream STAT3 (Fig. 7).

## Discussion

Electrical stimulation is involved with chondrogenic differentiation of MSCs in vitro, with reports showing positive effects on the induction of chondrogenesis and enhancement on matrix synthesis [19–21]. These studies employed traditional parameters, in which electrical stimulation was administered with low field strength over a period of 2–3 days, typically applied repeatedly during the differentiation period. The current study investigated the effects of nanosecond pulsed electric fields, administered to suspended MSCs over a short period (nanosecond pulses were applied five times at 1-s intervals), prior to chondrogenic induction and differentiation. Given the widely reported cytotoxic effects of nsPEFs, we first performed MSC apoptotic and viability tests using 16 nsPEF conditions with varying field strengths and durations. Although early apoptotic assays at 1 h showed most cells survived, cell viability assays taken at day 1 post-pulse indicated that nsPEFs with high field strengths and long durations significantly downregulated cell viability (Fig. 2). The effects of nsPEFs on MSC viability correlate well with the reported cytotoxic effects of nsPEFs, in which high-dose pulsing caused apoptosis and cell fragmentation. Mid-range pulse dosage might not incite plasma membrane permeability (thus impermeable to propidium iodide), but inducing the intracellular apoptotic pathway via cytochrome c and caspase activation can still affect cell viability [8, 23].

nsPEFs with varied parameters have different effects on the cells. According to physical models, nsPEFs with proper durations (10–300 ns) incur selective effects on intracellular structures and cell membranes based on the charging time constants of the membrane [3]. If the duration is longer than the charging time constants of the cell membrane (about 100 to 400 ns), electric fields will only affect the cell membrane. If the duration is shorter than the charging time constants of the cell membrane (about 100 to 400 ns), the electric fields will pass through the cell membrane and affect the intracellular structures. However, if the duration is far shorter than the charging time constants of organelle membranes (less than 10 to 30 ns), electric fields will probably not affect the cell membrane and the intracellular structures.

Subsequent cartilaginous gene expression analyses with the nsPEF-preconditioned MSCs subjected to chondrogenic differentiation led us to select nsPEFs of 10 ns at 20 kV/cm (condition A) and 100 ns at 10 kV/cm (condition B). These preconditioning parameters significantly upregulated expression levels of *ACAN*, *COL II*, and *SOX9*, with relatively low induction of *COL I* and *COL X* expression. The ability of these two nsPEF conditions to potentiate MSC chondrogenic differentiation was confirmed with increased deposition of matrix proteoglycan. Significantly, implantation of the nsPEF-preconditioned MSCs at the rat osteochondral defect enhanced cartilage regeneration (Fig. 4), indicating the long-term chondrogenic potential of the nsPEF-preconditioned MSCs. The implanted MSCs would proliferate in the defect and differentiate into chondrocytes to promote the repair of chondral defects [24, 25]. They also promoted regeneration via paracrine from cytokines and secreted growth factors [26].The nsPEF potentiation effect demonstrates the effectiveness of administering a single dose of pulse electromagnetic field at the onset of chondrogenic induction [27], indicating the importance of biophysical perturbation at the onset of chondrogenesis. To the best of our knowledge, this is the first report that identifies parameters of electrical preconditioning that potentiate chondrogenic differentiation of MSCs, and the effective application of the nsPEF-preconditioned MSCs to promote in vivo cartilage regeneration.

nsPEFs were shown to affect intracellular signaling pathways including the activation of the JNK, P38, ERK, and Wnt signaling pathways [13, 17, 28]. JNK was sensitive to external stimulus and was found to be activated by nsPEFs in Hela S3 cells [17, 29]. JNK, P38, and ERK belong to the mitogen-activated protein kinase (MAPK) family and have all been proven to affect chondrogenic differentiation of MSCs [30–32]. In addition, association with CREB, STAT3, and WNT/β-catenin pathways has been reported [32–34]. In this study, we demonstrate that JNK, CREB, and STAT3 phosphorylation were rapidly upregulated by nsPEF treatment of MSCs (Fig. 5), while ERK, P38, and β-catenin were not affected (Additional file 2 Figure S2). Inclusion of specific inhibitors to the JNK, CREB, and STAT3 pathways reduced activation of these pathways by nsPEFs, and concomitantly curtailed nsPEF-potentiated chondrogenic effect (Fig. 6), implying that nsPEF preconditioning promotes chondrogenic differentiation of MSCs through activation of JNK, CREB, and STAT3. Inhibition of JNK or CREB phosphorylation alone can partially downregulate the phosphorylation of STAT3 induced by nsPEFs (Fig. 5e), suggesting that JNK and CREB are upstream regulators of STAT3 signaling. Notably, the reduction of nsPEF-induced chondrogenesis by STAT3 inhibition was significantly more drastic than that by JNK or CREB inhibition alone, and was equivalent to the combined inhibition of JNK and CREB. STAT3, differentiation-related proteins, was found to be phosphorylated by the activation of the JNK signaling pathway [35]. Activation of STAT3 was found to positively regulate chondrogenic differentiation of MSCs by upregulation of *SOX9* [34, 36] and loss of STAT3 resulted in global embryonic decrease of *SOX9* expression [37]. CREB-binding protein (CBP/p300) was also associated with *SOX9* transcriptional activity and was shown to interact with *SOX9* in the cell nucleus during chondrogenesis [33, 38]. On the other hand, CREB could also activate downstream STAT3 signaling pathways, as reported in pancreatic cancer cell growth [39]. Similarly, STAT3 has been found to be phosphorylated by activation of the JNK signaling pathway [35]. Taken together, our results suggest that nsPEF preconditioning promotes chondrogenic differentiation of MSCs through activation of JNK and CREB, and is dependent on downstream activation of STAT3 (Fig. 7).

It remains unclear how nsPEF treatment resulted in the activation of intracellular signaling pathways. Administration of nsPEFs has shown to regulate intracellular calcium concentration [40]. nsPEF exposure of pancreatic cell caused formation of transient nanopores in the plasma membrane and organelle membranes with immediate increase in intracellular Ca^2+^ [41], which in turn could activate JNK [42]. Inducing the release of transient calcium by electrical stimulation was also reported to activate the CREB signaling pathway [43]. In our experiment, MSCs were suspended in PBS without Ca^2+^ to avoid the effects of extracellular Ca^2+^, and we found that the calcium inhibitor BAPTA-AM blocked the activation of CREB (Fig. 5c, d) and partially blocked the activation of JNK caused by nsPEFs (Fig. 5a, b). Thus, it is likely that nsPEF treatment might cause structural changes to organelle membranes and induce immediate release of intracellular Ca^2+^ that activates JNK and CREB (Fig. 7). The intracellular effect of nsPEFs is likely cell type-dependent. nsPEFs of condition B, used in this study, were shown to activate Wnt/β-catenin signaling in chondrocytes in our previous study [13] but did not affect Wnt/β-catenin signaling in nsPEF-treated MSCs (Additional file 2 Figure S2). This highlights the necessity to systematically characterize effective pulsing parameters, and the elicited intracellular activation, when applying nsPEFs to different cell types.

Taken together, our in vitro chondrogenic differentiation studies and in vivo cartilage repair studies demonstrate a unique approach of applying nsPEF preconditioning to potentiate chondrogenic differentiation of MSCs. Such a preconditioning approach is particularly attractive for the translational application of MSCs for cartilage repair, as MSCs can be preconditioned prior to implantation within minutes and thus circumvent the complication associated with on-site direct pulsing of tissues.

## Conclusion

Through histology, quantification of extracellular matrix proteins, and gene expression, it was found that nsPEF preconditioning with optimized parameters significantly enhanced chondrogenic differentiation of MSCs in vitro. Additionally, MSCs preconditioned with nsPEFs significantly enhanced cartilage regeneration in osteochondral defect models of rats in vivo. Activation of JNK, CREB, and STAT3 signaling pathways plays a role in this process.

## Additional files

Additional file 1: **Table S1.** Primer sequences used for qRT-PCR. Primers of COL I, COL II, COL X, ACAN, SOX9, GAPDH, and c-Jun. (DOCX 14 kb)

Additional file 2: **Figure S2.** nsPEF effect on Wnt/β-catenin, P38 and ERK signaling pathways. (A, B) western blot analysis for P38, p-P38, ERK, p-ERK, β-catenin. (C-G) protein quantitation of p-P38, p-ERK, β-catenin, *n* = 4. (TIF 2349 kb)

Additional file 3: **Figure S1.** Effect of nsPEFs on MSCs viability and gene expression. (A) Cell count as quantified by Kit-8 assay at day 3 after pulsing. *n* = 4 for each group. Cell count was expressed as fold of control (non-pulsed samples). *, *P* < 0.05; **, *P* < 0.01; ***, *P* < 0.001. (B) Gene expression level for c-Jun. (TIF 527 kb)

## Abbreviations

ACAN: Aggrecan
CCK-8: Cell Counting Kit-8
COL I: Type I collagen
COL II: Type II collagen
COL X: Type X collagen
CREB: cAMP-response element binding protein
DMEM: Dulbecco’s modified Eagle’s medium
H&E: Hematoxylin and eosin
JNK: c-Jun NH2-terminal kinase
MAPK: Mitogen-activated protein kinase
MSCs: Mesenchymal stem cells
nsPEFs: Nanosecond pulsed electric fields
PI: Propidium iodide
sGAG: Glycosaminoglycans
SOX: Sex-determining region Y-type high mobility group box
STAT3: Signal transducer and activator of transcription MAPK
TGF-β: Transforming growth factor beta
