# Supplementary material for: Nanosecond pulsed electric fields enhanced chondrogenic potential of mesenchymal stem cells via JNK/CREB-STAT3 signaling pathway
Source: Stem Cell Res Ther. 2019 Jan 24;10:45. doi: 10.1186/s13287-019-1133-0 (PMC6346554; doi:10.1186/s13287-019-1133-0)
Supplement: Supplementary file 1 — Table S1. Primer sequences used for qRT-PCR. Primers of COL I, COL II, COL X, ACAN, SOX9, GAPDH, and c-Jun. (DOCX 14 kb) [file 13287_2019_1133_MOESM1_ESM.docx]

| Gene product | Forward primers (5’-3’) | Reverse primers (5’-3’) |
| --- | --- | --- |
| COL I | CAGAACGGCCTCAGGTACCA | CAGATCACGTCATCGCACAAC |
| COL II | GAGAGGTCTTCCTGGCAAAG | AAGTCCCTGGAAGCCAGAT |
| COL X | CAGGTACCAGAGGTCCCATC | CATTGAGGCCCTTAGTTGCT |
| ACAN | CGAAACATCACCGAGGGT | GCAAATGTAAAGGGCTCCTC |
| SOX 9 | ATCAGTACCCGCACCTGCAC | CTTGTAATCCGGGTGGTCCTT |
| GAPDH | GTCATCCATGACAACTTCGG | GCCACAGTTTCCCAGAGG |
| c-jun | CCCCTGTCTCCCATCGACATG | TTGCAACTGCTGCGTTAGCAT |

**Additional File 1 Table S1. Primer sequences used for qRT-PCR.**
