# Supplementary figures and images for: Nanosecond pulsed electric fields enhanced chondrogenic potential of mesenchymal stem cells via JNK/CREB-STAT3 signaling pathway
Source: Stem Cell Res Ther. 2019 Jan 24;10:45. doi: 10.1186/s13287-019-1133-0 (PMC6346554; doi:10.1186/s13287-019-1133-0)

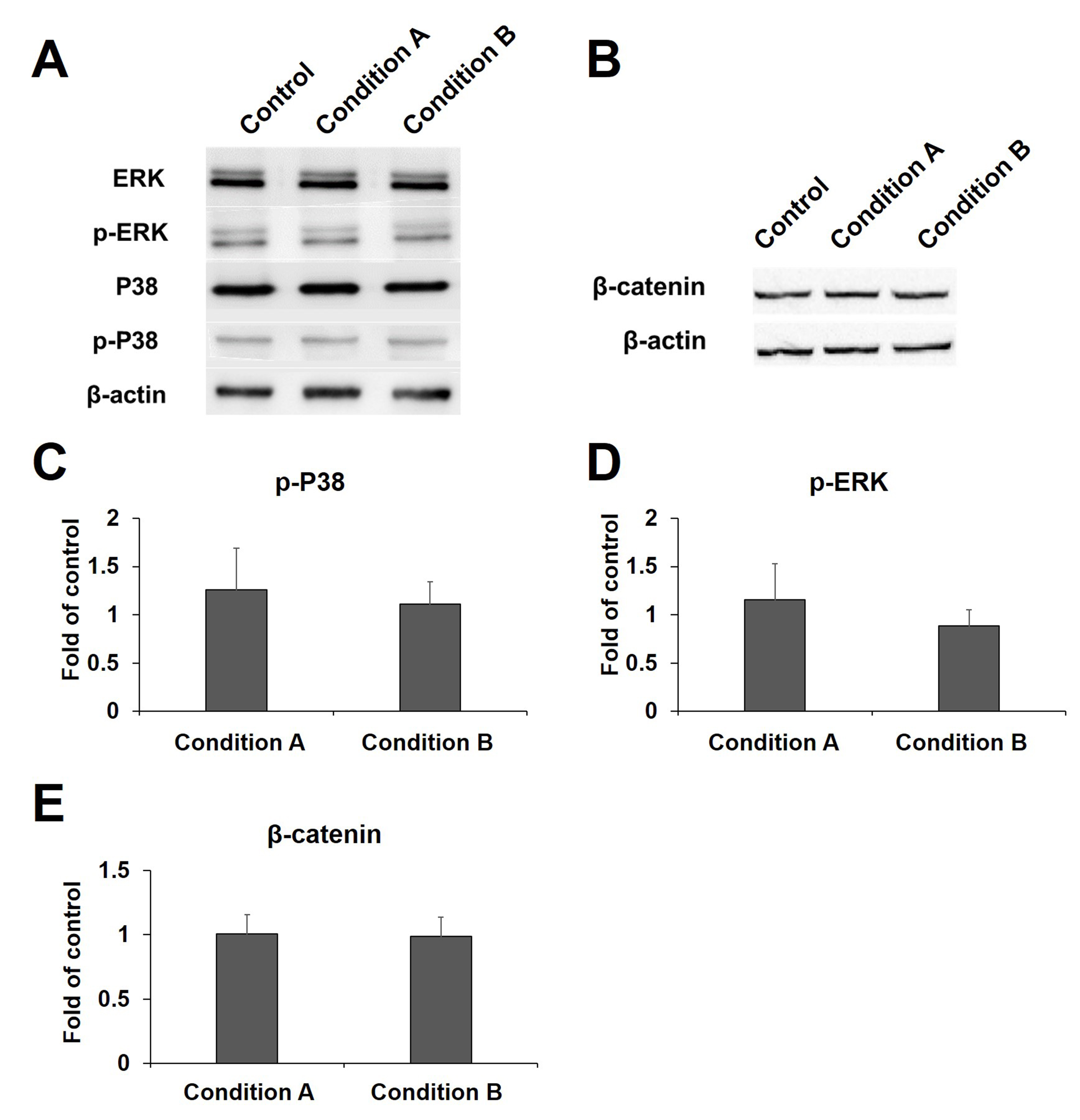

Supplement: Supplementary file 2 — Figure S2. nsPEF effect on Wnt/β-catenin, P38 and ERK signaling pathways. (A, B) western blot analysis for P38, p-P38, ERK, p-ERK, β-catenin. (C-G) protein quantitation of p-P38, p-ERK, β-catenin, n = 4. (TIF 2349 kb) [file 13287_2019_1133_MOESM2_ESM.tif]

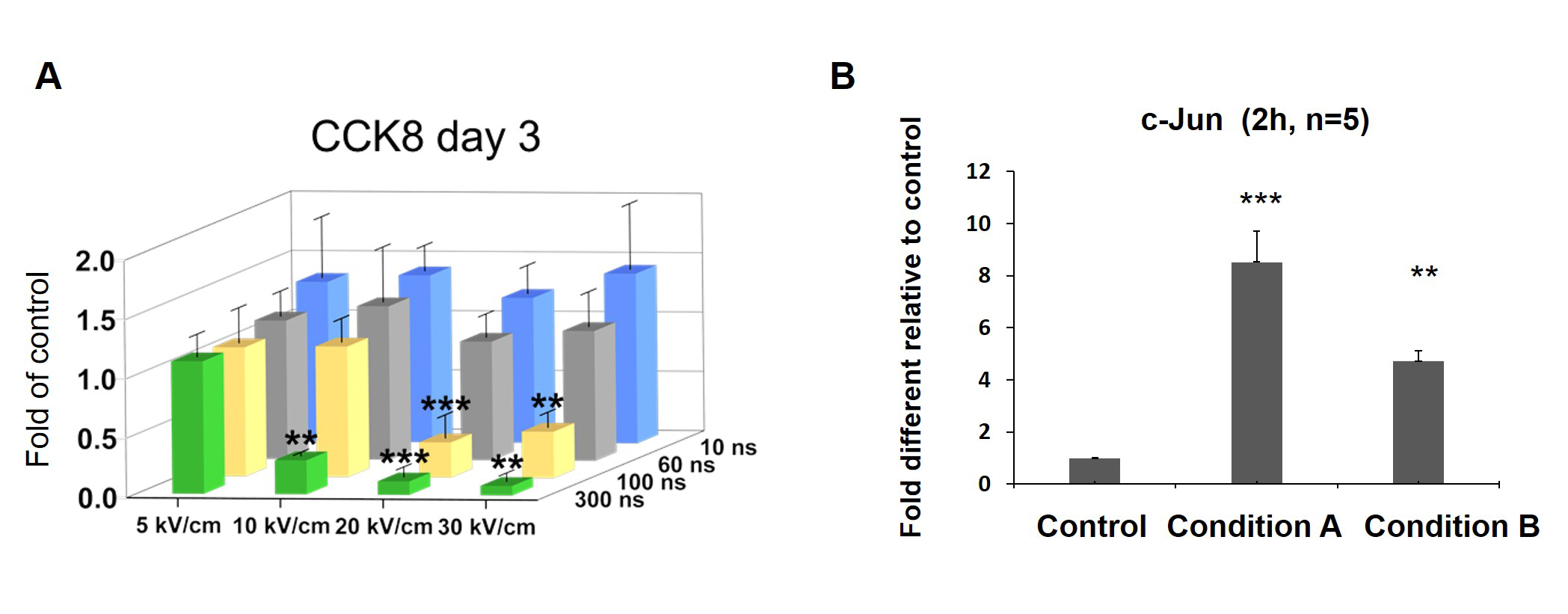

Supplement: Supplementary file 3 — Figure S1. Effect of nsPEFs on MSCs viability and gene expression. (A) Cell count as quantified by Kit-8 assay at day 3 after pulsing. n = 4 for each group. Cell count was expressed as fold of control (non-pulsed samples). *, P < 0.05; **, P < 0.01; ***, P < 0.001. (B) Gene expression level for c-Jun. (TIF 527 kb) [file 13287_2019_1133_MOESM3_ESM.tif]
